# Effect of Marble Dust on the Mechanical, Morphological, and Wear Performance of Basalt Fibre-Reinforced Epoxy Composites for Structural Applications

**DOI:** 10.3390/polym14071325

**Published:** 2022-03-24

**Authors:** Abhinay Singh Rajawat, Sanjeev Singh, Brijesh Gangil, Lalit Ranakoti, Shubham Sharma, Muhammad Rizal Muhammad Asyraf, Muhammad Rizal Razman

**Affiliations:** 1 Department of Mechanical Engineering, Maharana Pratap College of Technology, Gwalior 474406, Madhya Pradesh, India; abhinay.singh2002@gmail.com (A.S.R.); sanju141032@gmail.com (S.S.); 2 Mechanical Engineering Department, H.N.B. Garhwal University, Srinagar-Garhwal 246174, Uttarakhand, India; brijeshgangil@gmail.com; 3 Mechanical Engineering Department, Graphic Era Deemed to be University, Dehradun 248003, Uttarakhand, India; lalit_9000@yahoo.com; 4 University Center for Research & Development, Mechanical Engineering Department, Chandigarh University, Mohali 140413, Punjab, India; 5 Department of Mechanical Engineering, Main Campus-Kapurthala, IK Gujral Punjab Technical University, Kapurthala 144603, Punjab, India; 6 Institute of Energy Infrastructure, Universiti Tenaga Nasional, Jalan IKRAM-UNITEN, Kajang 43000, Selangor, Malaysia; asyrafriz96@gmail.com; 7 Research Centre for Sustainability Science and Governance (S.G.K.), Institute for Environment and Development (LESTARI), Universiti Kebangsaan Malaysia (U.K.M.), Bangi 43600, Selangor, Malaysia

**Keywords:** basalt fibre, marble dust, mechanical, SEM analysis, sliding wear

## Abstract

The reinforcement of natural fibre and fillers in polymer resin is the latest trend followed by research groups and industries for the development of sustainable composites. Basalt fibre and waste marble powder are naturally occurring substances used to enhanced polymer properties. The present research examined the effect of both basalt fibre and waste marble powder in epoxy resin. The hand lay-up method was employed to fabricate the composite and test for mechanical and wear behaviour. The tensile, flexural, and impact energy were enhanced up to 7.5 wt. % of WMP, and the Vickers hardness of epoxy enhanced every state of reinforcement of WMP. The specific wear rate was observed to be increased with the addition of WMP until 7.5 wt. %. Scanning electron microscopy was performed to examine the nature of fractured surface wear phenomena.

## 1. Introduction

The accelerating growth in natural fibre–reinforced polymer composites (NFRP) sphere confirms its importance for accomplishing end-user needs, particularly daily goods [1]. However, its low strength compared to synthetic fibre-reinforced composites limit its application to products, such as interior decoration materials for the household, automobile dashboards, chairs and tables, electrical equipment, piping, etc. [2,3,4,5]. The applied pressure and load are usually low to moderate [5,6,7,8,9,10]. In the present scenario, emissions of greenhouse gases and waste aggregation in landfills and aquatic environments due to processing of synthetic fibres have made the expansion of NFRP applications compulsory for heavy-duty materials for the development of sustainable and biodegradable composites to move ahead in the side lining of SFRPs [11]. 

Basalt, a type of mineral fibre, has received widespread acclamation in the last few decades due to the inherent characteristics, such as strength comparable to E-glass, a high modulus, excellent fatigue and shock-absorbing capability, and high resistance against corrosion and heat. It is cheap, readily available, does not release any toxic gas during processing, and is thus economically and environmentally stable [7,10]. Basalt fibre mainly contains oxides of silicon (52%) with a small percentage of aluminium, iron, calcium, magnesium, sodium, potassium, titanium, phosphorous, and manganese and, thus, qualifies as a suitable candidate for reinforcing in cement concrete [12,13,14]. 

Until now, the significance of reinforcing basalt fibre in polymers to improve the mechanical and thermal properties has not been much explored [15,16,17,18,19,20,21,22,23,24]. This is true for randomly oriented short basalt fibre polymer composites. Considering the recent literature, hybrid composites comprised of carbon/basalt/epoxy showed better fracture strain and tensile strain as compared to carbon fibre epoxy composites; however, the tensile properties were reduced [22,23,24,25,26,27,28,29,30,31]. These findings suggested that the arrangement of carbon/basalt fibre in the composite plays a crucial role in enhancing composites’ strength [32]. 

The tensile strength and Rockwell hardness of epoxy increased up to 47% and 50%, respectively, with 60% bidirectional basalt fibre reinforcement. In addition, more than 800% and 400% improvement were obtained in the value of impact energy and flexural strength, respectively [29]. Owing to its remarkable strengthening characteristics, it has been reported that basalt fibres composites were better in mechanical strength compared with glass fibre composites [13,16,22,33]. 

In many instances, polymer strengthening has been achieved by adding short basalt fibre, which improved the tensile strength but decreased impact strength [24]. In such cases, various fillers are added to compensate the later [34]. Numerous types of fillers, such as silicon carbide, dolomite powder, boron nitride, marble dust powder, etc., are added to toughen the polymers, thus, recognized as a crucial aspect in the strengthening, usually at low cost [6,35]. Although innumerable fillers are qualified to be selected as a filler, marble dust powder distinguishes itself due to availability, low-cost, and recyclability. In addition, its mechanical properties, low density, and high resistance against wear make it a competent filler substitute in a polymer [19]. 

The use of natural fibre and waste filler to fabricate sustainable material has become the priority of the research fraternity of polymer composites [15,25,26,27]. For instance, the incorporation of 20 wt. % marble dust waste in glass fibre-epoxy composite resulted in excellent mechanical properties. However, increasing the marble content beyond 20 wt. % reduced the strength [4,5]. 

At a fixed percentage of waste marble dust (15 wt. %), changing the bagasse fibre weightage to 7.5% improved the mechanical and physical properties of composites [31]. Not only the mechanical properties but also the wear performance, thermal characteristics, and fire retardancy of the composite were found to be enhanced by the inclusion of marble dust in fibre polymer composites [1,20,28]. 

In order to gain the maximum benefit of reinforcement, fibre and filler are hybridized together in one single matrix. For instance, Mostovoy et al. 2020 and Mostovoy et al. 2022 have performed dispersion of multiwalled carbon nano tubes in epoxy modified with γ-aminopropyltriethoxysilane (APTES) and found significantly enhancement of 194%, 137% 300% in bending stress, bending modulus and impact strength, respectively [17,18]. In another instance, Mostovoy at al 2021 reinforced carbon and glass fibre along with fillers of alumina oxide particles to fabricate hybrid composites by reinforcing in epoxy resin [18]. 

It was revealed that, at 3 wt. % aluminium oxide, the tensile strength of hybrid composite increased by the factor of 73%. Not only static behaviour but also the dynamic behaviour of Al_2_O_3_ filled carbon fibre–reinforced polymer composites can be significantly enhanced as fabricated via vacuum assisted resin infusion moulding (VARIM) as reported by Kaybal et al. 2018 [12]. Apart from nano filler and synthetic fibre, the hybridization of natural fibre brings fruitful results for the mechanical strength of the composite. Feng et al. 2020 fabricated kenaf/pineapple fibre-reinforced poly propylene hybrid composites and found that the tensile, flexural, and impact strengths were increased by 11%, 16% and 7%, respectively, as compared to single fibre composites [9].

The advantages of basalt fibres and marble dust in polymer composites as distinctly discussed earlier, are not one but many, and their combination in one single composite may further enhance the properties of polymer for the making of stiff, sustainable, and environmentally friendly composites with wider prospect of applications. The literature reveals that the basalt fibre is as strong as glass fibre and provides noteworthy improvement in the mechanical properties of polymers at low cost. 

In addition, waste marble powder is a hard particle available easily at low cost, which not only significantly improves the strength but also imparts hardness to the matrix material. The combination of both reinforcements may lead to the development of novel material, which can be used in low load structural applications. Therefore, the investigation attempts to develop short basalt fibre epoxy composite dispersed with waste marble powder (WMP). The fabricated hybrid composite was characterized in detail with physical (density and void content), mechanical (tensile, flexural, impact, and hardness), and wear behaviour. Microscopic analysis was also performed on the fractured sample to understand the mechanism of stress and mechanical closeness of the fibre/filler matrix.

## 2. Material and Fabrication

**Figure 1:**
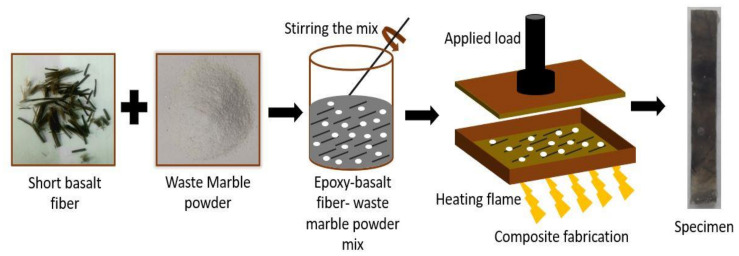
Schematic diagram of the fabrication of composite.

To fabricate the composite, short basalt fibres of length ranging from 10 to 15 mm were purchased from the local shop in Srinagar, Uttarakhand. Waste marble powder of mesh size 300 and density 2.68 g/cm^3^ was received from Dhaulpur, Rajasthan. Epoxy resin (LY5052) and hardener (HY5052) were procured from Amtech Ester Pvt. Ltd. (New Delhi, India), and its properties are shown in Table 1. The ratio of epoxy to hardener was taken as 100:10 to make the required matrix. The hand lay-up method was used to fabricate composites, as represented in Figure 1. Short basalt fibres and WMP were mixed with epoxy resin in a separate container. By manually stirring the mixture, homogeneous formation was achieved. Afterward, the mixture was poured into the mould and pressed at a maximum pressure of 2 MPa by the gradually applied load.

The mixture and the mould plate were separated by the mylar sheet and sprayed with silicon fluid to easily remove fabricated composite panels. The mould was heated by a flame at constant temperature of 150 °C for 5 min to release the air bubbles present in the mixture. The process was repeated for the fabrication of all the samples with varying WMP content. The weightage of basalt fibre was kept constant at 20 wt. %, whereas the WMP was varied in the proportions of 0, 2.5, 5, 7.5, and 10 wt. %. The composite designation and weight percentage of the reinforced material and resin are illustrated in Table 2. 

## 3. Composite Characterizations

Composite were characterized for physical, mechanical, and wear behaviour. Three respected tests were replicated for each composite, and the average values are provided in the study. Physical (density and void), properties were evaluated for the fabricated composites. The Archimedes principle was applied to find the experimental density, and the theoretical density was calculated by the relation given in Equation (1). Consequently, the void fraction was calculated by the normalization of theoretical density and the experimental density as given in Equation (2).

(1) $${\;\rho }_{\mathrm{th}}=\frac{1}{\frac{W_{m}}{\rho_{m}}+\frac{W_{b}}{\rho_{b}}+\frac{W_{\mathrm{wmp}}}{\rho_{\mathrm{wmp}}}\;}$$

(2) $$V_{f}=\frac{\rho_{\mathrm{th}}-\rho_{\exp}}{\rho_{\mathrm{th}}}$$

where ρ_th_ = theoretical density, W_m_, W_b_, and W_wmp_ are the weight fraction of matrix, basalt fibre, and waste marble powder, respectively. ρ_m_, ρ_b_, and ρ_wmp_ are the theoretical density of basalt fibre, waste marble powder, and matrix, respectively. As illustrated in Equation (2), V_f_ denotes void fraction and ρ_th_ and ρ_exp_ are the theoretical and experimental density of the sample.

ASTM D 3039 was adopted to determine the tensile properties of the specimen having dimension 150 mm × 15 mm × 4 mm whereas, flexural properties were determined as per ASTM D790-07 standard on a specimen having dimension 125 mm × 15 mm × 4 mm. Both tensile and flexural tests were performed on a digital Universal testing machine (UTM) at 3 mm min^−1^ of crosshead speed. Impact properties were determined with the help of digital impact testing machine as per ASTM E23 standard on sample size 55 mm × 10 mm × 10 mm having a 2 mm deep notch cut at an angle of 450. 

A Vickers hardness machine was employed to characterize the hardness of composites as per ASTM E-92 standard using diamond shape indenter provided with an apical angle of 136°. A gradual load of 1 kgf was applied on the specimen for an estimated time of 10 s. ASTM G-99-17 standard was implemented to examine the wear behaviour of the developed composite. Specimens of size 30 mm × 6 mm × 3.5 mm were forced against a steel disc of grade EN 32 under varying load and velocity. The specific wear rate was calculated by the relation as given in Equation (3). In the wear analysis, the normal load was varied from 10 to 40 N, and sliding velocity was varied from 3.5 to 7 m/s.

(3) $$W_{s}=\frac{\Delta m}{\rho_{\exp}\times T\times V_{s}\times F_{n}}$$

where Δm denotes the difference in mass before and after the wear, ρ_exp_ symbolizes the experimental density, T represents the time of wear, V_s_ is the sliding velocity, and F_n_ is the normal load. Finally, microscopic analysis was performed by imparting an electron beam of 8 kV of energy on the fractured specimen coated with a thin layer of gold thickness using SIGMA scanning electron microscopy (SEM) at a working distance of 10 mm.

## 4. Results and Discussion

### 4.1. EDX Analysis

**Figure 2:**
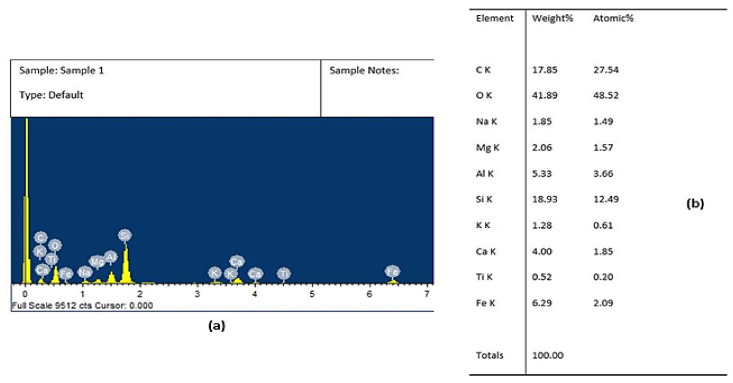
Showing (**a**) EDAX of basalt fibre and (**b**) percentage of elements in basalt fibre.

Energy-dispersive X-ray microanalysis (EDX) was performed on basalt fibre as illustrated in Figure 2a,b, which shows various elements, such as carbon, oxygen, sodium, calcium, potassium, etc. We found that basalt fibre consisted of a very high amount of silicon and carbon, followed by iron and aluminium. The weightage of oxygen was found to be maximum among all the elements.

### 4.2. Density and Voids

**Figure 3:**
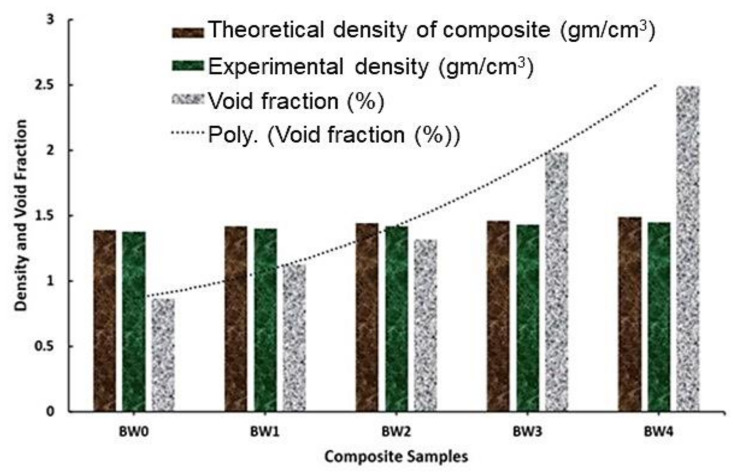
Density and voids of the fabricated composites.

As shown in Figure 3, the densities of the composites increased both theoretically and experimentally with the addition of WMP due to the replacement of low-density epoxy with high-density WMP. The theoretical density varied from 1.39 g/cm^3^ (0 wt. % of WMP) to 1.488 g/cm^3^ (10 wt. % of WMP) and the experimental density varied from 1.378 g/cm^3^ (0 wt. % of WMP) to 1.45 g/cm^3^ (10 wt. % of WMP). Likewise, the void fraction of the composites demonstrated an increasing trend as seen in Figure 3. 

This is due to the inadequate wettability of WMP in the epoxy resin coupled with the entrapment of air in the composite during the solidification [2]. However, the void present in the composite lies in the limiting range thus justifying the feasibility of the process. The composites were solidified with extra care to remove air bubbles, as the highest number of voids found in them was within the limiting range, namely 2.45% at 10 wt. % of WMP [8].

### 4.3. Mechanical Properties

**Figure 4:**
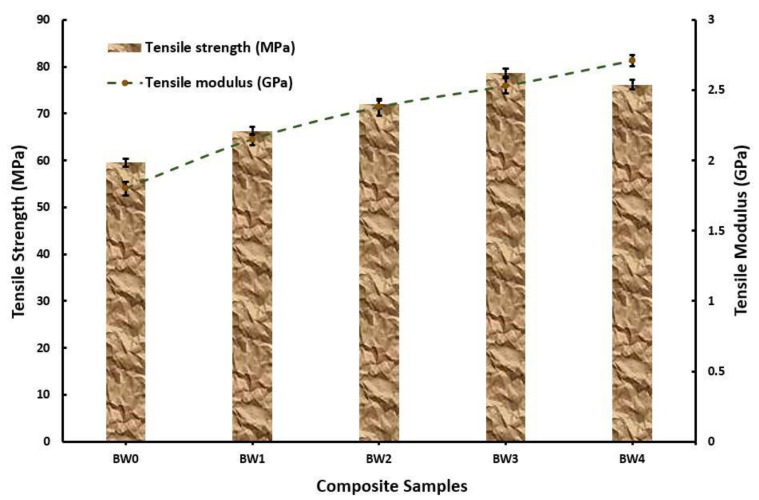
Tensile strength and modulus of the fabricated composites.

The tensile strength of the prepared composite samples is represented in Figure 4. The tensile strength obtained at 0 wt. % of WMP was 59.6 MPa, which was enhanced by 11. 2% upon addition of 2.5 wt. % WMP. The enhancement in the tensile strength continued until the wt. % of WMP reached 7.5, which was 78.7 MPa. The reason for the enhancement is majorly attributed to two factors viz: the reinforcement of strong basalt fibre and the addition of marble powder [35,36,37,38]. The intermetallic bonding with epoxy of basalt fibre provided strength to the composite whereas the WMP acted as a bridging agent between the basalt and WMP. However, a meagre reduction in the tensile strength was noticed at 10 wt. % of WMP due to the collection particles of WMP, which results in higher stress concentration at the fractured section. 

**Figure 5:**
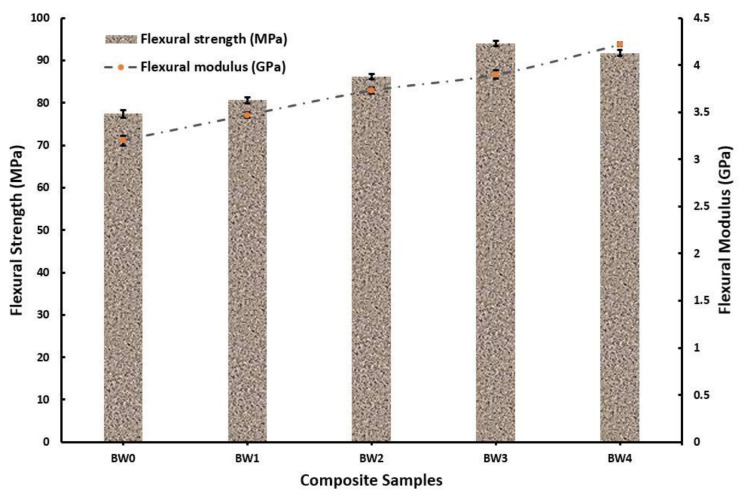
Flexural strength and modulus of the fabricated composites.

This enhancement of tensile strength reflected on the tensile modulus, which increased from 1.8 GPa at 0 wt. % WMP to 2.71 GPa at 10 wt. % WMP. The inclusion of WMP caused a higher amount of voids formation, resulting in dislocation cloud formation upon loading of specimen [27]. The flexural strength of the composite’s samples increased at a similar pace as observed in the case of tensile strength but at a higher magnitude as seen in Figure 5. Observations revealed that flexural strength of 77.4 MPa and 91.7 MPa was observed at 0 wt. % WMP and 10 wt. % WMP, respectively. However, the maximum flexural strength was obtained at 7.5 wt. % WMP. Similar findings were reported by Nayak, S.K., and Satapathy, A [19].

The flexural strength of the composite’s samples was found to be increased at similar pace as observed in case of tensile strength but at higher magnitude as seen in Figure 5. Observations revealed that flexural strengths of 77.4 and 91.7 MPa were observed at 0 wt. % WMP and 10 wt. % WMP, respectively. 

However, the maximum flexural strength was obtained at 7.5 wt. % WMP. The increase in the flexural strength was because of the shear behaviour WMP provided in the composite. Upon loading the specimen, the particles of WMP deformed in the lateral direction and transferred the shear forces to the basalt fibre, which led to more considerable deformation in the lateral direction, which consequently increased the load applied and delayed the time of fracture [4,5,39,40,41,42,43,44,45,46,47,48,49,50,51,52,53,54,55,56,57,58,59,60,61,62,63].

Figure 5 also reveals the flexural modulus, which increased from 3.2 GPa at 0 wt. % WMP to 4.22 GPa at 10 wt. % WMP. Since the flexural modulus depends on the lateral deformation of the composites, it may be the WMP inclusion by which the transfer of shear stress among the WMP, basalt and epoxy transpired easily leading to enhanced flexural strength. 

**Figure 6:**
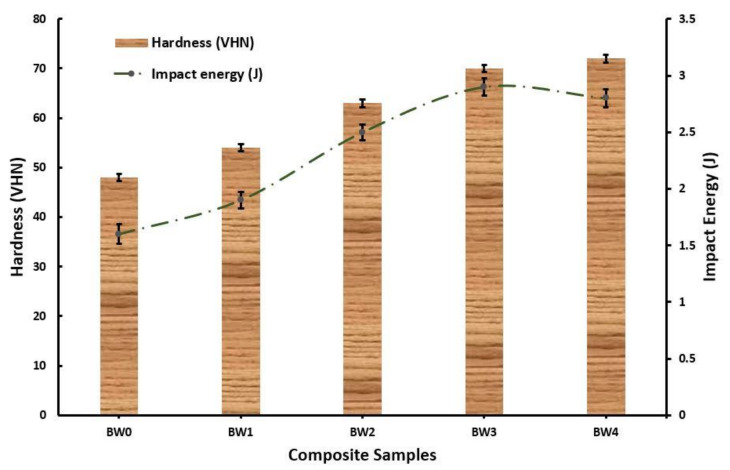
Hardness and impact energy of the fabricated composites.

The impact energy absorbed by the composite before fracture and the Vickers hardness number of the sample are shown in Figure 6. The impact energy at 0 wt. % WMP was observed as 1.6 J, which increased by the inclusion of WMP and found to be 2.9 J at 7.5 wt. % WMP. This happened due to the high load bearing capability of both basalt and WMP. WMP acted as a connecting chain between the basalt fibre and epoxy resin promoting transfer of stress, which led to high deformation before fracture [39,40,41,42,43,44,45,46,47,48,49,50,51,52,53,54,55,56,57,58,59,60,61,62,63,64,65,66,67,68,69]. Moreover, large amount of dislocation formation due to the inclusion of WMP in the composite resulted in higher energy absorption. However, the reduction in the impact energy at 10 wt. % WMP may be attributed to the accumulation of hard WMP particle at single cross section leading to formation of early crack. 

The Vickers hardness of the composite as shown in Figure 6 revealed that the addition of WMP in the basalt fibre epoxy composite is very much fruitful. The Vickers hardness number increased from 48 VHN at 0 wt. % WMP to 72 VHN at 10 wt. %, since WMP are the low dense hard particles and mostly accumulated at the surface during solidification. These hard particles when come in contact with the indenter offered high resistance as reported earlier [30], which in turn results in the higher Vickers hardness number.

### 4.4. Specific Wear Rate

Polymer composite materials are often subjected to applications where the phenomena of sliding wear prevail, such as artificial joints, cylinder liners, equipment of automobiles, contact bushes in electrical devices, etc. In order to widen the wear applications in dynamic conditions of hybrid composite material based on fibres and fillers, sliding wear analysis was conducted. 

**Figure 7:**
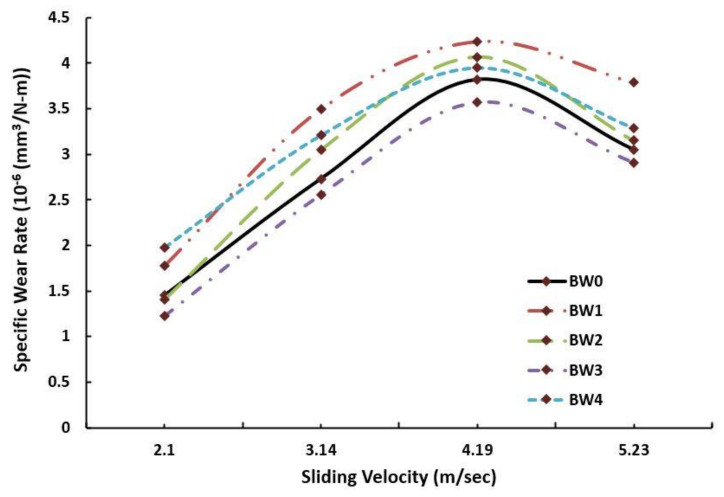
Specific wear rate vs. sliding velocity for the fabricated composites.

Figure 7 demonstrates the specific wear rate at different sliding velocity and constant normal load of 15 N. The sliding distance was kept fixed at 1000 m. Initially, at low velocity i.e., 2.1 m/s, the wear rate was found to be minimum for all the compositions with the BW3 samples showing the least wear rate among all, which was 1.23 × 10^−6^ mm^3^/N-m. As the velocity increased, the specific wear also increased due to the interaction of resin with the counter-rotating disc, and it continued to grow until the velocity reached 4.19 m/s. At this stage, the maximum specific wear was observed as 4.24 × 10^−6^ mm^3^/N-m for the sample BW1. Further increasing the velocity led to a decrease in specific wear rate for all the compositions. This reduction may be attributed to the resistance offered by both WMP and basalt fibre against the wear.

Both basalt fibre and WMP are hard surfaces and are difficult to wear. After subsequent increases in the wear, when these reinforcements came in contact with the steel disc, the specific wear decreased [70,71,72,73,74,75,76,77,78,79,80,81,82,83,84,85,86,87]. 

**Figure 8:**
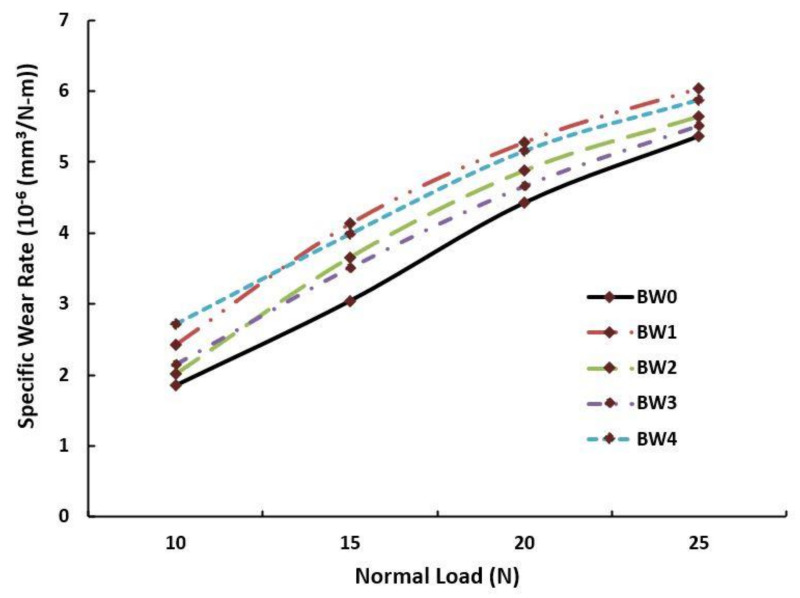
Specific wear vs. normal load for the fabricated composites.

Unlike the case of sliding wear, the observation for specific wear at different normal load conditions was constantly increasing as observed in Figure 8. At the initial stage i.e., at 10 N, the specific wear was minimal; however, as the load increased, the composite material was forced against the steel disc giving rise to friction between the mating surfaces and ultimately led to heat generation. This caused melting of the resin, deformation, breaking of fibres, and disintegration of WMP [59,60,61,62]. 

At higher loads, this phenomenon prevailed and caused higher specific wear. The maximum specific wear was found to be 6.04 × 10^−6^ mm^3^/N-m at 25 N for the BW1 composition, and the minimum was observed as 1.85 × 10^−6^ mm^3^/N-m at 10 N for the BW0 composition. 

### 4.5. Fractography of the Composites

**Figure 9:**
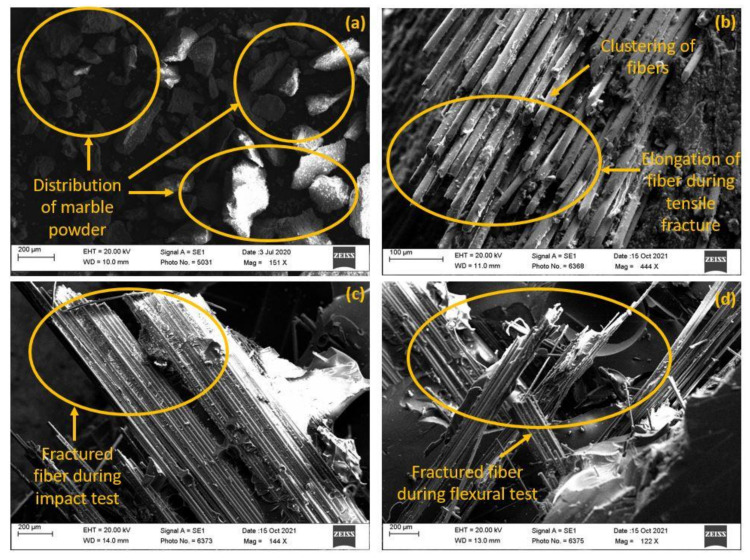
(**a**) Waste marble powder granules, Fractographs of BW4: (**b**) tensile specimen, (**c**) impact specimen, and (**d**) flexural specimen.

The Fractographs of tensile, impact, and flexural specimens are depicted in Figure 9. The distribution of WMP in the composite can be seen in Figure 9a. We observed that the size of WMP particles differs and varies as per the range stated earlier. This distribution was also observed to be almost homogeneous to comply with the mixing phenomena that was performed during the fabrication [57,58,59,60,61,62,63,64]. 

Figure 9b shows the deformation mechanism and clustering of fibre at one section occurred due to the fibre pull out and fibre breakage during the tensile test. The uneven sectional breakage of the fibre can be seen in Figure 9c, which occurred during the impact loading, may be attributed to the non-homogenous distribution of fibre at one section leading to stress concentration and crack growth. In Figure 9d, the breakage of fibres occurred at lateral direction due to the applied bending load. Moreover, during the flexural test, the fractured zone experienced deformation of fibre along the lateral direction coupled with the brittle fracture due to the stress concentration arisen due to inhomogeneity of the fibre and matrix [43,44,45].

### 4.6. Wear Analysis

**Figure 10:**
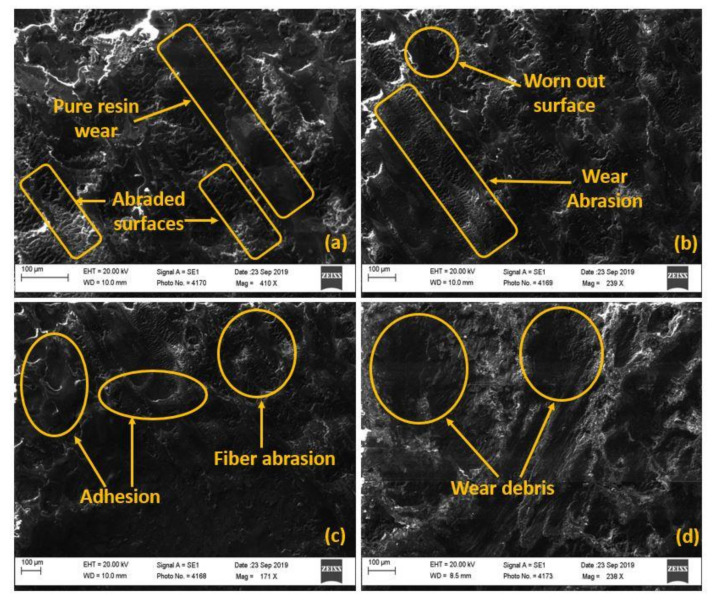
Images showing wear phenomena at various marble filler loading: (**a**) BW1, (**b**) BW2, (**c**) BW3, and (**d**) BW4.

The wear examination of different composition samples is depicted in Figure 10. We found that the surface experienced various phenomena viz: adhesion, abrasion, wear debris, etc. For instance, at 2.5 wt. % WMP, the sample suffered fibre abrasion coupled with pure resin wear as confirmed by Figure 10a. This is clear since the percentage reinforcement was low; consequently, there was less interaction of WMP and the counter-rotating disc. Worn out surfaces and wear abrasion were observed at 5 wt. % WMP i.e., Figure 10b might have occurred due to the rigidness induced by the incorporation of WMP leading to the detachment of the outer surfaces from the core. 

The adhesion phenomena dominated at 7.5 wt. % of WMP (Figure 10c) since the composition formed a slurry kind of mix at elevated temperature, and instead of forming fragments, it attached to the surfaces. The formation of wear debris that was observed at 10 wt. % WMP might result from an accumulation of WMP leading to high stress concentration [61,62,63,64,88,89,90,91,92,93,94,95,96,97,98,99,100,101,102,103,104].

## 5. Conclusions

In this study, epoxy composites with fixed basalt fibre (20 wt.%) and changing marble dust filler (0,2.5, 5, 7.5, and 10 wt.%) were developed, manufactured, and tested for mechanical and wear behaviour. The following conclusions can be drawn from this study:

i. Generally, the properties, such as the tensile strength, flexural strength, hardness, and impact strength, increased with the increase in marble dust proportion. This might be due to the fact the composite was strengthened by the intermetallic bonding of basalt fibre with epoxy, while the WMP served as a bridging agent between the basalt and the WMP. Overall, the BW3 composite gave the best results.
ii A small reduction in properties was observed at 10 wt. WMP due to the collection of particles of WMP resulting in a higher stress concentration at the fractured section. Fractograph images also confirmed the deformation mechanism and clustering of fibres.
iii In the context of sliding wear, specific wear at different speeds and normal loads was steadily observed. Due to the interaction between the resin and the counter-rotating disc, the specific wear increased as the velocity increased, and it continued to rise until the velocity reached 4.19 m/s. As the velocity increased further, the specific wear rate of all compositions decreased. This reduction may be due to the wear resistance of WMP and basalt fibres.
iv At higher loads, this phenomenon prevailed and caused higher specific wear. The maximum specific wear was found to be 6.04 × 10^−6^ mm^3^/N-m at 25 N for the BW1 composition, and the minimum was observed as 1.85 × 10^−6^ mm^3^/N-m at 10 N for the BW0 composition.

## Figures and Tables

**Table 1:** Properties of Epoxy E-21.

Appearance	Colourless to Light Yellow Liquid.
Epoxy equivalent weight	185–194 G/EQ
Viscosity 25 °C	11,000–14,000 cP
Hydrolysable Chlorine	0.05% max.
Epoxide value	5.15–5.40
Density at 25 °C	1.16 g/cm^3^
Moisture content	0.1% max.
ECH content	10 ppm max.
Non-volatile content	100%
Flash point	>150 °C

**Table 2:** Weightage of reinforcement and resin.

Composition (wt. %)			
Designation	Basalt	Epoxy	Waste Marble Powder
BW0	20	80	0
BW1	20	77.5	2.5
BW2	20	75	5
BW3	20	72.5	7.5
BW4	20	70	10

## Data Availability

No data were used to support this study.
